# First-principles study on the electronic and optical properties of AlSb monolayer

**DOI:** 10.1038/s41598-023-37081-5

**Published:** 2023-06-19

**Authors:** Mohammad Ali Mohebpour, Meysam Bagheri Tagani

**Affiliations:** Computational Nanophysics Laboratory (CNL), Department of physics, University of Guilan, P. O. Box 41335-1914, Rasht, Iran

**Keywords:** Nanoscale materials, Chemical physics, Condensed-matter physics

## Abstract

Using density functional theory and many-body perturbation theory, we systematically investigate the optoelectronic properties of AlSb monolayer, which has been recently synthesized by molecular beam epitaxy [ACS Nano 2021, 15, 5, 8184–8191]. After confirming the dynamical stability of the monolayer, we analyze its electronic properties at different levels of theory without (PBE, HSE03, HSE06) and with (G$_0$W$_0$, GW$_0$, and GW) electron-electron interaction. The results show that AlSb monolayer is a semiconductor with a direct quasiparticle band gap of 1.35 eV while its electronic structure is dominated by spin-orbit coupling. Also, we study the optical properties of the monolayer by solving the Bethe–Salpeter equation. In this regard, the effects of spin-orbit coupling, electron–electron correlation, and electron–hole interaction on the optical spectrum of the monolayer are evaluated. Based on the highest level of theory, the first bright exciton is found to be located at 0.97 eV, in excellent agreement with the experimental value (0.93 eV). Moreover, the exciton binding energy, effective mass, and Bohr radius are obtained 0.38 eV, 0.25 m$_0$, and 6.31 Å, respectively. This work provides a better understanding of the electronic, optical, and excitonic properties of AlSb monolayer and may shed light on its potential applications.

## Introduction

Over the past two decades, graphene^1^ has aroused the interest of researchers due to its incredible properties such as ultrahigh carrier mobility ($\sim$ 2 $\times$ 10$^5$ cm$^2$/Vs)^2^, superhigh thermal conductivity (3–5 $\times$ 10$^3$ W/mK)^3^, ultrahigh transparency ($\sim$ 98%)^4^, and very large Young’s modulus ($\sim$ 1 TPa) and ideal strength ($\sim$ 100 GPa)^5^. Despite these fabulous features, the absence of a band gap in graphene has restricted its potential applications in nanoelectronic devices. Hence, researchers have had to explore new two-dimensional (2D) materials. Nowadays, we have a large family of 2D materials with a variety of different characteristics such as ultralow lattice thermal conductivity^6,7^, large absorption coefficient^8–10^, and large exciton binding energy^11,12^.

Group III–V binary monolayers are one of the most important classes of 2D materials with attractive properties such as high stability, high carrier mobility, suitable band gap, and strong spin-orbit coupling^13–16^. They are one of the hottest topics for experimental and theoretical researches owing to their potential applications in nanoelectronics^17,18^, optoelectronics^19,20^, photovoltaics^21,22^, photocatalysis^22,23^, and thermoelectricity^24,25^. Recently, Lucking et al.^26^ predicted that the group III–V semiconducting monolayers stabilize in a double-layer honeycomb (DLHC) structure. Following that, Qin et al.^27^ successfully synthesized the DLHC AlSb monolayer on a graphene-covered SiC(0001) substrate using molecular beam epitaxy. They exposed that AlSb monolayer is more stable than its bulk structure and has a band gap of 0.93 eV, which is smaller than 1.6 eV of the bulk. After this achievement, a theoretical work^28^ explored the electronic, mechanical, and optical properties of AlSb monolayer. They obtained reasonable values for the elastic constants and Young’s modulus while their optoelectronic results are based on the simplest level of density functional theory without considering many-body effects, which play an important role in 2D materials. Also, Dong et al.^29^ investigated the electronic properties of AlSb monolayer using the first-principles calculations coupled with Bethe–Salpeter equation. They reported an excitonic instability as a result of the larger exciton binding energy than the corresponding energy gap. Besides, Bafekry et al.^30^ studied the effects of substitutional doping and vacancy point defects on the electronic and magnetic properties of AlSb monolayer and revealed that most of the vacancy defects and substitutional dopants change the behavior of the monolayer from semiconducting to metallic. Despite all these works, the need for a comprehensive description of optoelectronic properties of AlSb monolayer considering many-body effects is feeling.

In this paper, we investigate the optoelectronic properties of the AlSb monolayer by employing density functional theory coupled with many-body perturbation theory. First, we show the dynamical and thermodynamical stabilities of the monolayer. Then, we explore its electronic properties at different levels of theory without (PBE, HSE03, HSE06) and with (G$_0$W$_0$, GW$_0$, and GW) considering electron–electron interaction. We find that AlSb monolayer is a semiconductor with a direct quasiparticle band gap of 1.35 eV. Following that, we study the optical properties of the monolayer by solving the Bethe–Salpeter equation on top of the GW eigenvalues. In this regard, we examine the effects of spin-orbit coupling, electron–electron correlation, and electron–hole interaction on the optical spectrum. We find that these interactions highly dominate the intrinsic optoelectronic properties of the monolayer. Our work provides a better understanding of the electronic, optical, and excitonic properties of AlSb monolayer and may attract attention to the group III–V monolayers.

## Computational details

The first-principles calculations were performed based on the density functional theory (DFT) using the Vienna ab-initio simulation package (VASP)^31^. The generalized gradient approximation developed by Perdew–Burke–Ernzerhof (GGA-PBE)^32^ was adopted for the estimation of the exchange-correlation potential. The projector augmented wave (PAW) method was used for the description of the electron–ion interaction. The total energy was converged with respect to energy cutoff and k-point mesh. The energy cutoff was chosen to be 450 eV. The Brillouin zone (BZ) was integrated with a 12 $\times$ 12 $\times$ 1 $\Gamma$-centered k-point mesh. The total energy was minimized until the energy difference between two successive steps became less than 10$^{-7}$ eV. The DFT-D2 method of Grimme was employed to include the van der Waals (vdW) interaction^33^. The convergence criterion for the Hellmann–Feynman forces was taken to be 10$^{-3}$ eV/Å. The vacuum space was selected to be 20 Å  to eliminate the interactions between periodic images.

To show the characteristics of the bonds in AlSb monolayer, the charge transfer between atoms was determined on the basis of the Bader analysis^34^. To ensure the dynamical stability of the monolayer, the phonon dispersion calculation was performed using the finite displacement method as implemented in the PHONOPY code^35^. We employed 3 $\times$ 3 $\times$ 1 supercells with 5 $\times$ 5 $\times$ 1 k-point meshes in the calculation of the second-order interatomic force constants.

The many-body perturbative calculations were done using the GW approximation. The calculations were performed at three different levels of self-consistency from the single-shot version of the GW (i.e. G$_0$W$_0$) to the partially self-consistent ones (i.e. GW$_0$ and GW). Upon the convergence test, a set of 200 virtual bands was selected. The energy cutoff for the response function was set to be 200 eV. The vacuum space was imposed to be 30 Å. The k-point mesh was chosen to be 12 $\times$ 12 $\times$ 1. In the partially self-consistent GW, 4 iterations were sufficient to reach an excellent convergence. The G$_0$W$_0$ band structure was interpolated using the maximally localized Wannier functions as implemented in the WANNIER90 code^36^. The number of Wannier bands was selected to be 16, and the $sp^3$ hybrid orbitals were selected for initial projections. The optical properties were determined by solving the Bethe–Salpeter equation (BSE) on top of the GW eigenvalues, considering the 10 highest valence bands and the 10 lowest conduction bands. The BSE calculations were simplified by excluding the anti-resonant part of the BS Hamiltonian using the Tamm–Dancoff approximation (TDA)^37^. The same input parameters were utilized for when spin-orbit coupling (SOC) is included in the calculations. It should be noted that the DFT calculations include SOC and the Kohn–Sham states obtained by the calculations are used to perform a fully relativistic G$_0$W$_0$ calculation. Indeed, Green’s function (G) is calculated using two-component spinor Kohn–Sham states. Note that AlSb monolayer is not spin-polarized and the final results are spin-independent parameters.

## Results and discussion

### Structural and electronic properties

**Figure 1:**
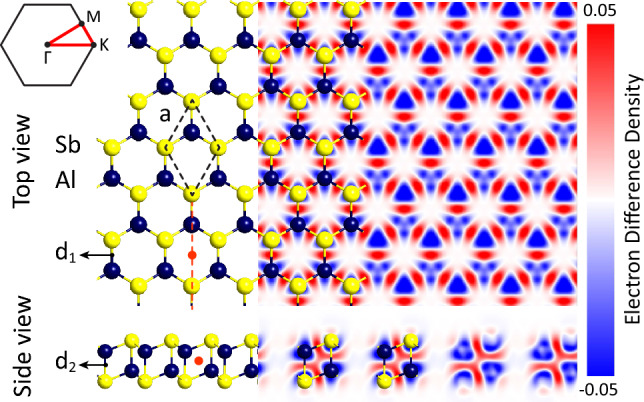
Top and side views of AlSb monolayer along with the electron density difference map. The unit cell and Brillouin zone are shown by black dashed and solid lines, respectively. The inversion symmetry center is shown by a red circle and the mirror symmetry is shown by a red dashed line.

Figure 1 illustrates the top and side views of AlSb monolayer. This monolayer has a hexagonal lattice similar to the $\beta$-antimonene bilayer with AA stacking^38^. Both of the Al and Sb atoms form four bonds with nearly $sp^3$ hybridization. There is an inversion symmetry center between the two sublayers of AlSb and a mirror symmetry in the y–z plane. Upon full relaxation, the lattice constant of the monolayer is found to be $a=4.29$ Å and the bond lengths are $d_1=2.71$ and $d_2=2.86$ Å, respectively. The bond lengths are slightly larger than the sum of the covalent radii of Al (1.24 Å) and Sb (1.40 Å) atoms, showing the ionic bonding of AlSb monolayer. The structural parameters are listed in Supplementary Table S1, in excellent agreement with previous works^27,28^.

The electron density difference map of AlSb monolayer is also displayed in Fig. 1, where the red and blue regions signify charge accumulation and depletion, respectively. Obviously, the maximum accumulation is located at the center of the in-plane bonds between Al and Sb atoms ($d_1$). Moreover, there is a minor electron accumulation at the center of the out-of-plane bonds between Al and Sb atoms ($d_2$), which accommodates the electron deficiency of Al atoms and is responsible for the stability of the structure. On the other hand, the maximum depletion is observed around Al atoms. These are in line with the Bader charge analysis, which approves that 1.42 charge is transferred from Al to Sb atoms, showing the ionic bonding character. The charge transfer from Al to Sb atoms is reasonable since the electronegativity of Sb (2.05) is larger than that of Al (1.61).

Supplementary Figure S1 indicates the phonon dispersion spectrum of AlSb monolayer. Apparently, there is no imaginary vibrational frequency in the first BZ, confirming the dynamical stability of the monolayer. To validate the thermodynamical stability, we calculated the cohesive energy ($E_c$) as below:

1 $$\begin{aligned} E_{c(f)}=\dfrac{~~2E_{Al}+2E_{Sb}-E_T~~}{4}, \end{aligned}$$

where $E_T$ is the total energy of the monolayer and $E_{Al}$ ($E_{Sb}$) is the ground state energy of isolated Al (Sb) single atom. As given in Supplementary Table S1, the cohesive energy is 4.49 eV/atom, larger than those of $\beta$-antimonene (2.84 eV/atom)^39^, $\beta$-arsenene (2.99 eV/atom)^40^, and phosphorene (3.48 eV/atom)^41^, showing the high stability of AlSb monolayer. By considering the single-atom energy achieved from the bulk aluminum and antimony, the formation energy ($E_f$) is similarly found to be 0.19 eV/atom, which approves that the reaction leading to the formation of AlSb monolayer is exothermic.

The electronic band structures and density of states of AlSb monolayer are shown in Supplementary Fig. S2a. As it is clear, the PBE level predicts that AlSb monolayer is a semiconductor with a direct band gap of 0.1 eV at the $\Gamma$ point. The valence and conduction band edges are parabolically dispersed, which shows free charge carriers. The valence band maximum (VBM) has double degeneracy. There is a conduction band extremum at the M point, which has higher energy than the conduction band minimum (CBM) by 0.35 eV, providing an opportunity to reach the bands convergence via strain^6,42^. The calculated band gap is in line with the previously reported gap (0.13 eV)^26^. By including a fraction of the exact exchange energy in the DFT formulation, the size of band gap is corrected. In other words, the HSE03 and HSE06 functionals estimate the band gap to be 0.51 and 0.66 eV, respectively. The shapes of the band structures at these levels are similar to that of the PBE level as shown in Supplementary Fig. S2a.

From Supplementary Fig. S2a, it can also be seen that the valence band is mostly contributed by Sb atoms while the conduction band is mainly dominated by Al atoms. More specifically, the orbital-decomposed band structure given in Supplementary Fig. S2b shows that the Sb-$p_y$ and Al-*s* states have the highest contribution in the VBM and CBM, respectively. No considerable contribution from the Al-*p* and Sb-*s* states appears near the VBM and CBM. These are completely in line with the shapes of wave functions at the VBM and CBM as represented in Supplementary Fig. S2c. At the VBM, the wave function is shaped like a dumbbell, distributed along y-direction, while the center of dumbbell is on the Sb atom, showing the participation of the Sb-$p_y$ orbitals. At the CBM, the wave function is spherically symmetric and centered on the Al atom, showing the contribution of the Al-*s* orbitals. By and large, one can say that the ground state interband transition originates mainly from the Sb-*p* to Al-*s* states.

To show the anisotropic band dispersions, we calculated the orientation-dependent effective mass and Fermi velocity. As shown in Supplementary Fig. S3a, the effective mass of holes reaches the maximum value of 0.196 m$_0$ at 30$^{\circ }$, 90$^{\circ }$, 150$^{\circ }$, 210$^{\circ }$, 270$^{\circ }$, and 330$^{\circ }$ while reduces to 0.153 m$_0$ at 0$^{\circ }$, 60$^{\circ }$, 120$^{\circ }$, 180$^{\circ }$, 240$^{\circ }$, and 300$^{\circ }$. In other words, the effective mass periodically fluctuates in the range of 0.153–0.196 m$_0$. Hence, it is highly dependent on the orientation. For electrons, an opposite trend is observed and the anisotropic behavior is stronger. The effective mass of electrons reaches the maximum and minimum values of 0.293 and 0.182 m$_0$ at 0$^{\circ }$ and 90$^{\circ }$, respectively. This anisotropy is seen more clearly from the Fermi velocity as shown in Supplementary Fig. S3c,d. The Fermi velocity of holes is predicted to be around 0.12 and 0.42 $\times$ 10$^5$ m/s along the 0$^{\circ }$ and 90$^{\circ }$ directions, respectively. While for electrons, they are 8.52 and 5.61 $\times$ 10$^5$ m/s. Supplementary Fig. S3e,f illustrates the 2D energy map of the highest valence band and lowest conduction band and also the asymmetric band dispersion in different directions, which is in line with the anisotropic effective mass and Fermi velocity.

**Figure 2:**
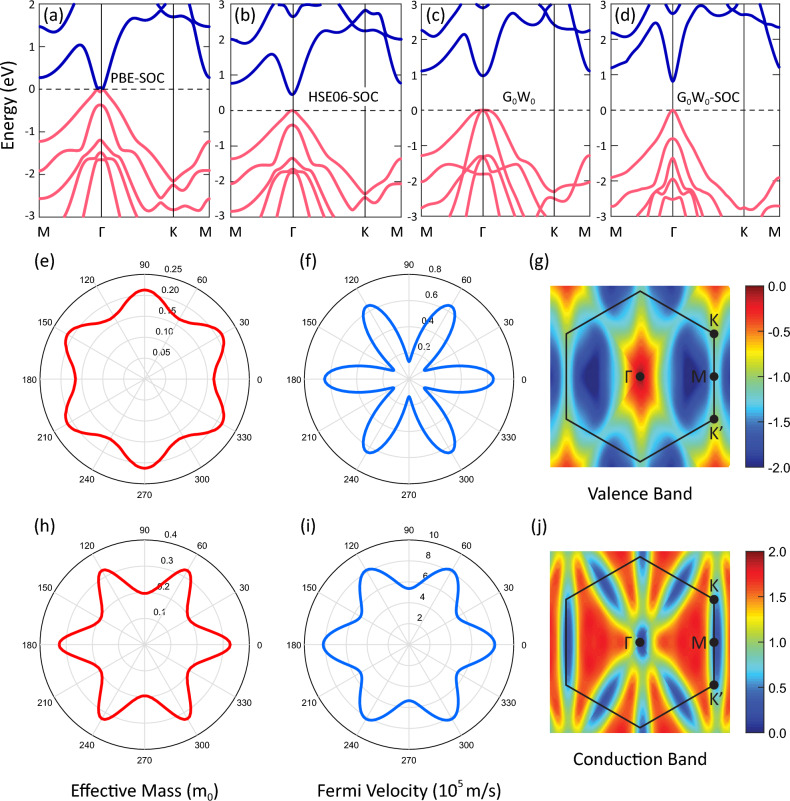
Band structures of AlSb monolayer at the (**a**) PBE-SOC, (**b**) HSE06-SOC, (**c**) G$_0$W$_0$, and (**d**) G$_0$W$_0$-SOC levels of theory. Orientation-dependent effective mass and Fermi velocity for holes (**e**,**f**) and electrons (**h**,**i**) along with the 2D energy map of the highest valence band (**h**) and lowest conduction band (**j**) considering SOC interaction.

**Table 1:** QP and gaps calculated for AlSb monolayer at different levels with and without SOC interaction in the unit of eV.

SOC	PBE	HSE03	HSE06	G$_0$W$_0$	GW$_0$	GW	HSE06-G$_0$W$_0$
Without	0.10	0.51	0.66	1.16	1.34	1.62	1.62
With	0.01	0.39	0.45	0.71	–	–	1.35

When SOC interaction is included at the PBE level, the band dispersion changes a lot and the band gap is almost closed ($E_g \sim 0.01$ eV). Interestingly, a Mexican hat dispersion appears in the highest valence band and lowest conduction band as shown in Fig. 2a. The Mexican hat will be more obvious when the band structure is plotted along the high symmetry points of $\Gamma$-K-M-$\Gamma$. Also, in the presence of SOC, the VBM and CBM are shifted from the $\Gamma$ point to the (0.01, 0.01, 0.0) point. Hence, one can say that SOC dominates the band structure. By considering SOC at the HSE06 level, the band gap increases to 0.45 eV while the Mexican hat dispersion disappears as shown in Fig. 2b. Also, SOC eliminates the degeneracy of the VBM at the $\Gamma$ point and opens a small gap of 0.4 eV between the heavy and light hole subbands. However, it cannot remove the double degeneracy of each energy state, which originates from the inversion symmetry center of the structure. Generally speaking, one can say that the main impact of SOC is in the valence band, which is made of the hybridization of the Sb-$p_x$ and Sb-$p_y$ orbitals. The conduction band is not considerably affected by SOC, owing to the predominant participation of Al-*s* orbitals. Such behavior was reported for transition metal dichalcogenides, where the conduction band edge is made of the $d_{z^2}$ orbitals and remains spin-degenerate whereas the valence band edge is split^43^.

As mentioned above, the energy bands are doubly degenerated when SOC is included. This comes from the inversion symmetry of the geometry structure. To show the relation between the inversion symmetry center and the double degeneracy of bands, we calculated the band structure of a conceptual model of AlSb monolayer. We broke the inversion symmetry of the structure by replacing the Al and Sb atoms in one sublayer. As shown in Supplementary Fig. S4, the bands are completely separated and the Zeeman-type splitting is observed, which is due to the combination of SOC and inversion symmetry breaking. In fact, we exposed the reason behind the double degeneracy of each energy state with SOC.

We also went beyond the simple DFT by performing the GW calculations, which include electron–electron interaction. The quasiparticle (QP) band gap achieved from many-body GW calculations depends on a few input parameters including the number of virtual bands, k-point mesh, thickness of vacuum space ($L_z$), energy cutoff, and iteration of the self-consistent loop. Therefore, we initially tested the convergence of the QP band gap at the G$_0$W$_0$ level with respect to the (a) number of virtual bands using $L_z = 20$ Å  and 6 $\times$ 6 $\times$ 1 k-point mesh; (b) vacuum space using 40 virtual bands and 6 $\times$ 6 $\times$ 1 k-point mesh; and (c) k-point mesh using 40 virtual bands and $L_z = 20$ Å. As represented in Supplementary Fig. S5a, 200 virtual bands are necessary to reach the convergence threshold of $\sim$ 10$^{-2}$ eV. Such fast convergence is attributed to the absence of highly localized states in the conduction band. The convergence with respect to the vacuum space is achieved after 30–35 Å, which is due to the long-range nature of the Coulomb interaction. But, because of the excessive memory requirement for a dense k-point mesh, 30 Å is the largest vacuum space we could computationally afford. We similarly understand that a 12 $\times$ 12 $\times$ 1 k-point mesh is sufficient to converge the QP band gap by $\sim$ 10$^{-1}$ eV. Our results also prove that 4 iterations are enough to reach the criterion of 10$^{-4}$ eV during updating the QP eigenvalues (see Supplementary Fig. S5d). For the response function, we set the energy cutoff to be 200 eV because the band gap is the same if a higher cutoff (300 eV, $\Delta$ $E_g$ $\sim$ 10$^{-2}$ eV) is used. For the BSE calculations, we considered the 10 highest valence bands and the 10 lowest conduction bands, which are enough to converge the optical spectrum at energies below 8 eV^44^. Considering a higher number of valence and conduction bands up to 15 may change the shape of the optical spectrum but at higher energies, which is not important for us in this work.

Figure 2c depicts the QP band structure of AlSb monolayer at the G$_0$W$_0$ level. As it is seen, the general shape of the band structure is similar to those achieved from the PBE and hybrid functionals in the absence of SOC shown in Supplementary Fig. S2a. The valence and conduction band edges are still located at the $\Gamma$ point. However, the inclusion of electron–electron interaction leads to a constant shift of the energy bands. The unoccupied bands are moved to higher energies while the occupied bands are shifted down to lower energies. As a result, the G$_0$W$_0$ level predicts the QP band gap to be 1.16 eV, which is larger than those obtained by the PBE and hybrid functionals. The calculated value is in good agreement with the G$_0$W$_0$ band gap (1.35 eV) reported by Dong et al.^29^. Such a slight difference might arise from the use of different computational codes and pseudopotentials. Also, it could be due to the synergic effect of a larger number of bands and a denser k-point mesh in their G$_0$W$_0$ calculations. They performed the G$_0$W$_0$ calculations with 312 virtual bands, 50 Ry for the plane wave cutoff, 12 Ry for the response function cutoff, and an 18 $\times$ 18 $\times$ 1 k-point mesh using the QUANTUM ESPRESSO code interfaced with YAMBO code. Surprisingly, they did not consider the vdW interactions, which mostly lower the band gap in 2D vdW materials. Updating the QP eigenvalues in the Green’s function (i.e. GW$_0$) increases the band gap of the monolayer up to 1.34 eV. Also, at the GW level, the QP band gap is found to be 1.62 eV. The QP band gaps calculated at different levels, follow the order of PBE < HSE03 < HSE06 < G$_0$W$_0$ < GW$_0$ < GW, which is similar to that reported for graphene derivatives by Karlicky et al.^45^.

By switching on SOC at the G$_0$W$_0$ level, the band gap of the monolayer reduces to 0.71 eV, which agrees very well with that (0.74 eV) reported by Dong et al.^29^. The band gap reduction with SOC has also been reported for bulk AlSb^46^. As shown in Fig. 2d, except for the size of band gap, the G$_0$W$_0$-SOC band structure highly resembles the HSE06-SOC one, including the band dispersion and the direct gap characteristic. It is a little different from the PBE-SOC band structure since there is no camel-back-like dispersion at the edges of the bands. Meanwhile, it is completely different from the PBE and G$_0$W$_0$ ones due to the strong effects of SOC. By and large, we found that the larger the band gap is, the stronger the strength of SOC will be. To better compare the levels of theory, we listed all the QP band gaps calculated with and without SOC in Table 1. Because the GW$_0$-SOC and GW-SOC calculations require high computational power and add nothing new to this work, we skipped these calculations.

Figure 2e,h shows the orientation-dependent effective mass of holes and electrons considering SOC. Similar to those calculated without SOC, the effective mass of holes has the maximum value at 30$^{\circ }$, 90$^{\circ }$, 150$^{\circ }$, 210$^{\circ }$, 270$^{\circ }$, and 330$^{\circ }$ while is minimum at 0$^{\circ }$, 60$^{\circ }$, 120$^{\circ }$, 180$^{\circ }$, 240$^{\circ }$, and 300$^{\circ }$, showing an anisotropic band dispersion. The effective mass of holes fluctuates in the range of 0.165–0.213 m$_0$. For electrons, an opposite trend is observed. The effective mass of electrons remains in the range of 0.196–0.327 m$_0$. Overall, one can say that the effective mass of carriers are predicted to be slightly larger with SOC. From Fig. 2f, the Fermi velocity of holes is estimated to be 0.65 and 0.13 $\times$ 10$^5$ m/s along the 0$^{\circ }$ and 90$^{\circ }$ directions, respectively. These are completely different from those calculated without SOC due to the considerable effect of SOC in the valence band edge. From Fig. 2i, the Fermi velocity of electrons is predicted to be 8.20 and 5.35 $\times$ 10$^5$ m/s along the 0$^{\circ }$ and 90$^{\circ }$ directions, respectively. These values are almost identical to those calculated without SOC because the conduction band edge is not considerably affected by SOC, owing to the predominant participation of Al-*s* orbitals. Figure 2g,j shows that the anisotropy of the bands still goes on in the presence of SOC. It is still in line with the anisotropic effective mass and Fermi velocity.

### Optical properties

**Figure 3:**
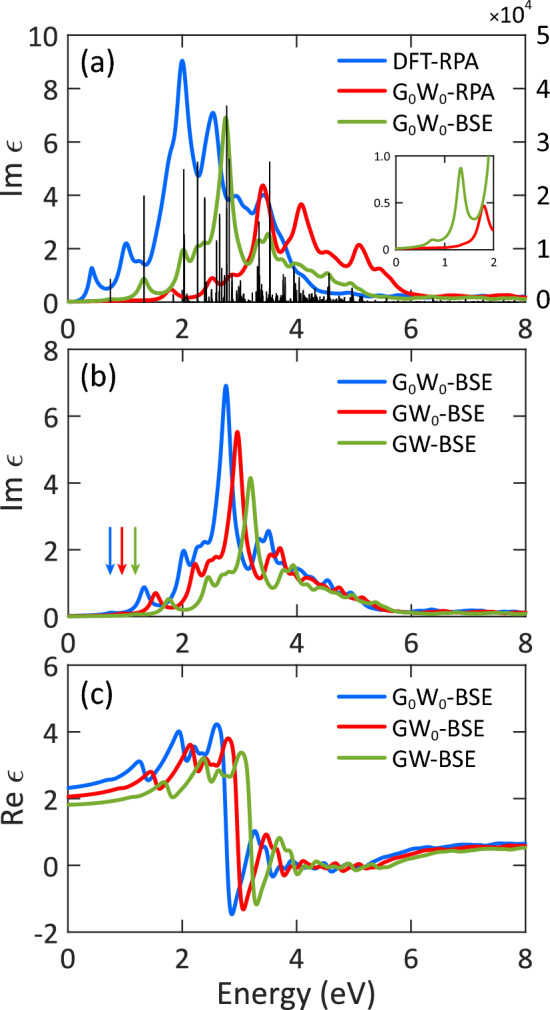
(**a**) Imaginary part of the dielectric function of AlSb monolayer at different levels of theory: DFT-RPA, G$_0$W$_0$-RPA, and G$_0$W$_0$-BSE. The black bars indicate the oscillator strength of the optical transitions. The inset shows a magnified model of the optical spectra. (**b**) Imaginary and (**c**) real parts of the dielectric function of AlSb monolayer under different degrees of self-consistency in the GW.

In this section, we investigated the optical properties of AlSb monolayer by solving the Bethe–Salpeter equation on top of the GW eigenvalues (the so-called GW-BSE), which accounts for both electron–electron and electron–hole interactions. To show the effects of many-body interaction, we calculated the optical spectra using the random-phase approximation (RPA), where the mentioned interactions are excluded. Owing to the symmetric geometry structure of AlSb monolayer, the optical spectra are perfectly isotropic for light polarized along the x-direction (E $\parallel$ x) and y-direction (E $\parallel$ y). This is why the results are only reported for x-direction. Figure 3a displays the imaginary part of the macroscopic dielectric function of AlSb monolayer at three levels including DFT-RPA, G$_0$W$_0$-RPA, and G$_0$W$_0$-BSE. As can be seen, the optical spectrum at the DFT-RPA level is characterized by five main peaks centered on 0.36, 1.01, 2.00, 2.54, and 3.42 eV. The first peak originates from a direct transition from the highest valence band (Sb-$p_y$ orbitals) to the lowest conduction band (Al-*s* orbitals) at the $\Gamma$ point. The inclusion of electron–electron interaction (G$_0$W$_0$-RPA) leads to a significant blueshift in the optical spectrum. This blueshift is a consequence of the self-energy correction. Moreover, the intensity of the peaks is greatly reduced with electron–electron interaction. Since the imaginary part of the dielectric function is directly related to the light absorption, one can say that the absorption is reduced in the presence of electron–electron interaction. When electron–hole interaction (G$_0$W$_0$-BSE) is included, the entire spectrum is pushed back, showing a cancellation effect. At this level, the overall shape of the spectrum is totally different. However, the main effect of electron–hole interaction is the advent of a tiny peak at 0.76 eV, which is not present at the G$_0$W$_0$-RPA level. This peak originates from excitonic effects. We plotted the oscillator strength of optical transitions as black bars in Fig. 3a. As can be seen, the oscillator strength of this tiny peak has a considerable magnitude ($\sim$ 4330), therefore, it corresponds to a bright exciton.

In Supplementary Fig. S6, we plotted the coefficients of the exciton wave function for the first bright exciton along the high symmetry lines in reciprocal space, where the radius of the circle reflects the magnitude of a particular electron-hole pair contribution. It is clear that the first bright exciton is exclusively composed of the transitions between the topmost valence and lowest conduction states at the $\Gamma$ point. Indeed, the most important part of the BZ is responsible for the first excitonic peak in the optical spectrum. This peak, the so-called optical gap, locates at 0.76 eV, which is smaller than the related QP band gap (1.16 eV), indicating a bound exciton with a binding energy of 0.40 eV. Such high exciton binding energy is attributed to the localization of exciton wave functions in real space and the weak screening environment^47^. The obtained optical gap is smaller than the experimental value (0.93 eV)^27^ owing to the lack of self-consistency in the G$_0$W$_0$, but it is in line with the 0.82 eV reported previously^29^.

Due to the considerable dependence of the G$_0$W$_0$ on the DFT-PBE starting point, we investigated the effects of different degrees of self-consistency in the GW as illustrated in Fig. 3b. Apparently, updating the QP eigenvalues in Green’s function only leads to a blueshift in the optical spectrum while maintaining its shape. At this level (GW$_0$-BSE), the optical gap is estimated to be 0.96 eV with an exciton binding energy of 0.38 eV. The obtained optical gap is in excellent agreement with the experimental gap (0.93 eV)^27^. It should be noted that this agreement could also due to the exclusion of SOC interaction. The obtained exciton binding energy is representative of bound excitons, showing the high stability of the excitonic states under thermal dissociation, making AlSb monolayer a promising candidate for optoelectronic devices. This value is close to that of graphyne (0.4 eV)^48^, and smaller than those of MoS$_2$ (0.96 eV)^49^ and graphane (1.6 eV)^50^, as the latter two monolayers have much larger QP band gaps of 2.8 and 5.4 eV, respectively.

Similarly, updating both the Green’s function and the screened Coulomb potential leads to a blueshift ($\sim$ 0.23 eV) as well, showing a larger self-energy correction in the GW compared to those in the G$_0$W$_0$ and GW$_0$. At the GW-BSE level (see Fig. 3b), the optical gap is found to be 1.19 eV, which is slightly overestimated compared to the experimental value^27^. One possible reason for this overestimation is the missing SOC interaction. At this level, the exciton binding energy is 0.43 eV.

Figure 3c exhibits the real part of the dielectric function. As expected, increasing the degree of self-consistency results in a blueshift in the optical spectrum, and consequently, a decrease in the static dielectric constant. The static value is 2.32, 2.06, and 1.81 at the G$_0$W$_0$-BSE, GW$_0$-BSE, and GW-BSE levels, respectively. The exclusion of electron–electron and electron–hole interactions (DFT-RPA, Data not shown) increases the dielectric constant up to 4.03, which seems to be overestimated. At all three levels, negative values appear, showing the metallic character of AlSb monolayer at these ranges of the electromagnetic spectrum.

**Figure 4:**
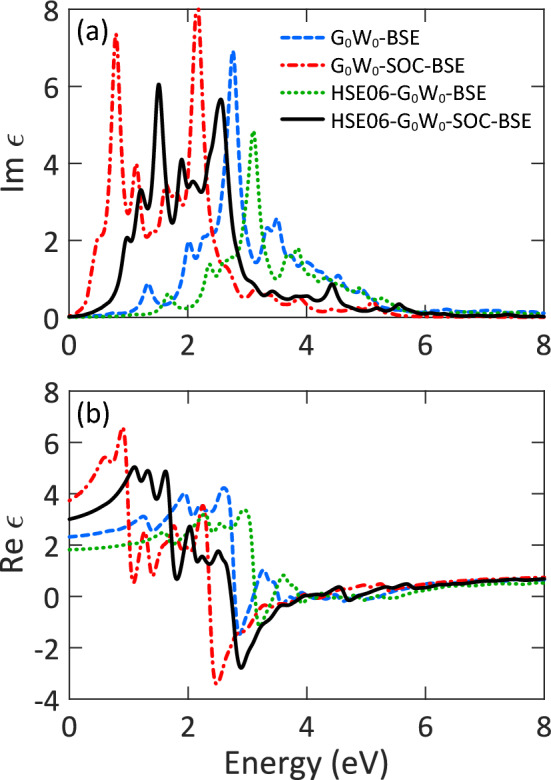
(**a**) Imaginary and (**b**) real parts of the dielectric function of AlSb monolayer at different levels of theory with and without SOC interaction.

**Table 2:** Optical gaps (Exciton binding energies) calculated for AlSb monolayer at different levels of theory with and without SOC interaction in the unit of eV. The experimental optical gap is 0.93 eV^27^. The HSE06-G$_0$W$_0$ level with SOC is in great agreement with the experiment.

SOC	G$_0$W$_0$	GW$_0$	GW	HSE06-G$_0$W$_0$
Without	0.76 (0.40)	0.96 (0.38)	1.19 (0.43)	1.10 (0.52)
With	0.52 (0.19)	–	–	0.97 (0.38)

We estimated the effective mass ($\mu$) and Bohr radius ($a_x$) of the ground state exciton based on:

2 $$\begin{aligned} \mu= & {} \frac{E_b}{R_h}\times \varepsilon _r^2\times m_0, \end{aligned}$$

3 $$\begin{aligned} a_x= & {} \frac{\varepsilon _r}{\mu }\times a_h \times m_0, \end{aligned}$$

where $R_h$ is the Rydberg constant (13.6 eV), $\varepsilon _r$ the dielectric constant, $m_0$ the electron rest mass, and $a_h$ the Bohr radius (0.529 Å). The effective mass of exciton is 0.158, 0.119, and 0.104 m$_0$ at the G$_0$W$_0$-BSE, GW$_0$-BSE, and GW-BSE levels, respectively. The Bohr radius of exciton is 7.75, 9.19, and 9.24 Å, respectively, which are smaller than those of MoS$_2$ ($\sim$ 10 Å)^51,52^, ZnO ($\sim$ 23 Å)^53^, and group IV–VI binary monolayers ($\sim$ 10–53 Å)^54^. A smaller Bohr radius means the electron and hole are restricted in a narrower area, therefore, they have stronger interaction. Owing to the large exciton binding energy and large exciton Bohr radius compared to the Al-Sb bond length, one can conclude that the excitons in AlSb monolayer have the characteristics of both the Wannier–Mott and Frenkel excitons.

**Figure 5:**
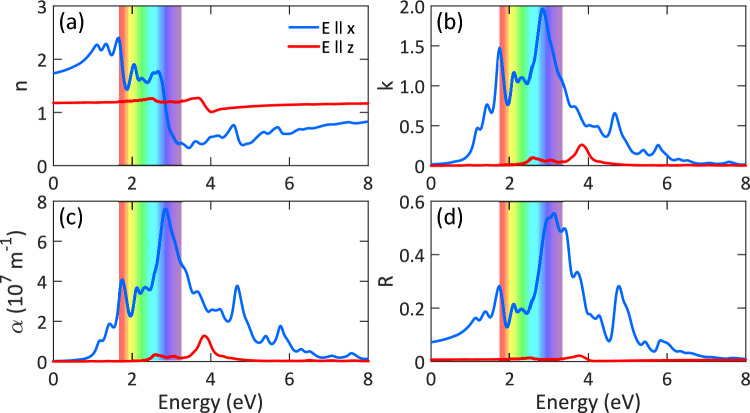
Optical properties of AlSb monolayer: (**a**) refractive index, (**b**) extinction coefficient, (**c**) absorption spectrum, and (**d**) reflectance for light polarized along the x-direction (E $\parallel$ x) and z-direction (E $\parallel$ z) considering SOC interaction.

**Figure 6:**
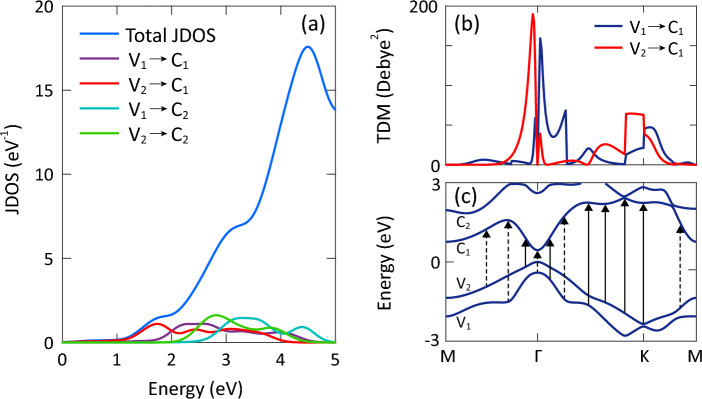
(**a**) Total and partial joint density of states, (**b**) transition dipole moment, and (**c**) electronic band structure of AlSb monolayer considering SOC interaction. The allowed and forbidden transitions are shown by solid and dashed arrows, respectively.

In this part, we investigated the effects of SOC on the optical spectrum of the monolayer. We knew that the G$_0$W$_0$-SOC calculations are performed on top of the PBE-SOC eigenvalues and eigenstates. And the PBE-SOC level highly underestimates the band gap of the monolayer as given in Table 1. To avoid the overscreening effects caused by an underestimated band gap, the G$_0$W$_0$-SOC calculations were performed on top of the HSE06-SOC eigenvalues and eigenstates. In other words, owing to the high computational cost of the GW$_0$-SOC and GW-SOC calculations, we solved the gap problem by using the HSE06-SOC eigenvalues and eigenstates as a starting point for the G$_0$W$_0$-SOC calculations as suggested and validated in the Refs.^45,55^. Figure 4a indicates the imaginary part of the dielectric function of AlSb monolayer with and without SOC. As expected, the inclusion of SOC at the G$_0$W$_0$-BSE level results in a redshift (0.24 eV) in the optical spectrum and splitting of the peaks. The splitting is attributed to the double degeneracy of energy bands in the presence of SOC. This level estimates the optical gap and exciton binding energy to be 0.52 and 0.19 eV, respectively. The obtained optical gap is 0.41 eV smaller than the experimental value owing to the overscreening effects caused by an underestimated band gap. And the obtained exciton binding energy seems to be underestimated. On the contrary, the HSE06 functional individually leads to a blueshift (0.34 eV) in the G$_0$W$_0$-BSE spectrum while conserving its shape. But, the HSE06-G$_0$W$_0$-SOC-BSE (HG$_0$SB) gives the proper optical spectrum owing to the synergic effects of SOC and HSE06. At this level, the first excitation energy is found to be 0.97 eV, in great agreement with the experiment^27^. This value is close to that obtained by the GW$_0$-BSE calculation without SOC. However, the shape of spectrum is noticeably different. Also, from the difference between the QP band gap and optical gap, the exciton binding energy is calculated to be 0.38 eV, which is close to those calculated without SOC. By and large, one can say that SOC strongly dominates the excitonic and optical properties of the monolayer. The final optical gap is slightly overestimated because the calculations were performed with a relatively coarse k-point mesh. To better compare the levels of theory, we listed all the optical gaps and binding energies calculated with and without SOC in Table 2.

Aside from a similar discussion for the real part of the dielectric function, from Fig. 4b, one can see that the static dielectric constant reaches 3.00 at the HG$_0$SB level. Accordingly, the effective mass and Bohr radius of exciton are obtained 0.251 m$_0$ and 6.31 Å, respectively. Indeed, with SOC, the effective mass (Bohr radius) is estimated to be larger (smaller), showing the crucial role of SOC in AlSb monolayer.

We calculated the other optical coefficients of AlSb monolayer at the HG$_0$SB level as it provides the correct optical gap and presents an optimum description of the optical spectrum. Figure 5a illustrates the refractive index of AlSb monolayer for light polarized along the x- and z-direction. Obviously, for the x-direction, the light is less refracted for energies higher than 3 eV. Specifically, the refractive index is less than 1.0 for energies higher than 3 eV, which means that AlSb monolayer is a highly transparent material at this range of the electromagnetic spectrum because its refractive index is smaller than that of glass ($\sim$ 1.52). The maximum (minimum) value of the refractive index is found to be 2.39 (0.33) at 1.64 (3.44) eV while the static value of the refractive index is 1.74. For the z-direction, as expected, the refractive index is almost independent of the photon energy and stays constant at 1.18. From Fig. 5b, it is seen that the extinction coefficient of AlSb monolayer increases quickly with increasing photon energy up to 2.84 eV, then, it decreases steadily. The maximum value of the extinction coefficient is found to be 1.96 at 2.84 eV. This means that at this energy, the photons will be absorbed very fast. As shown in Fig. 5c, the absorption threshold ($\alpha > 10^6$ m$^{-1}$) is located at 0.98 eV, precisely where the imaginary part of the dielectric function shows the first peak. Similar to the extinction coefficient, the absorption reaches its maximum value of 7.61 $\times$ 10$^7$ m$^{-1}$ at 2.84 eV in the visible area. After 2.84 eV, the optical absorption decreases drastically with increasing photon energy while shows oscillatory behavior. The mean value of absorption within the visible area (1.63–3.26 eV) is 4.56 $\times$ 10$^7$ m$^{-1}$, which is desired for optoelectronic applications. From Fig. 5d, the maximum reflectance is found to be 55% at 3.11 eV while the average value of reflectance in the visible area is less than 30%. Except for the slight peak at 4 eV, the nearly zero absorption and reflection confirm that AlSb monolayer is a transparent material for light polarized along the z-direction. This is due to the depolarization effect for light polarization perpendicular to the plane.

To see the contribution of inter-band transitions in optical absorption, we provide the total and partial joint density of states (JDOS) of AlSb monolayer in Fig. 6a. Obviously, the total JDOS increases rapidly with increasing energy up to 4.49 eV, then, it decreases gradually. More specifically, it shows two shoulders at 2.00 and 3.25 eV, which are correlated with transitions at the M point. The partial JDOS indicates that the optical absorption in the range of 1–2 eV is mainly dominated by transitions from the highest valence band to the lowest conduction band (i.e. V$_2$ $\rightarrow$ C$_1$). At 2 eV, the same contribution is observed from the V$_1$ $\rightarrow$ C$_1$ transitions. Also, the V$_1$ $\rightarrow$ C$_1$ and V$_2$ $\rightarrow$ C$_2$ transitions have the highest contribution in the range of 2.00–2.55 eV and 2.55–3.40 eV, respectively, where a weak contribution from the V$_2$ $\rightarrow$ C$_1$ transitions is observed. Figure 6b shows the probability of transitions between two states. Obviously, the value of transition dipole moment (TDM) is zero for most of the V$_2$ $\rightarrow$ C$_1$ transitions along the $\Gamma$ $\rightarrow$ M path. These transitions are called forbidden dipole moments. Meanwhile, the value of TDM is non-zero for these transitions near the $\Gamma$ point, which is why they are known as allowed transitions. It is also found that the V$_1$ $\rightarrow$ C$_1$ and V$_2$ $\rightarrow$ C$_1$ transitions are forbidden at the $\Gamma$ point while are allowed at the K point. Overall, one can say that the most probable transitions are located near the $\Gamma$ point. Figure 6c exhibits the most important forbidden (dashed arrows) and allowed (solid arrows) transitions.

## Conclusion

In summary, we study the electronic and optical properties of AlSb monolayer using density functional theory coupled with many-body perturbation theory. After confirming the ultrahigh stability of the monolayer by cohesive energy and phonon dispersion, we explore its electronic properties at different levels of theory without (PBE, HSE03, HSE06) and with (G$_0$W$_0$, GW$_0$, and GW) considering electron–electron correlation. The results show that AlSb monolayer is a semiconductor with a direct band gap of 1.35 eV. The optical spectrum achieved from the solution of the Bethe–Salpeter equation shows the first bright exciton of the monolayer to be located at 0.97 eV, which is in excellent agreement with the experimental value (0.93 eV). Accordingly, the exciton binding energy, effective mass, and Bohr radius are calculated to be 0.38 eV, 0.25 m$_0$, and 6.31 Å, respectively. We find that spin-orbit coupling, electron–electron correlation, and electron–hole interaction strongly dominate the intrinsic optoelectronic properties of the monolayer. Our work could attract more attention to the two-dimensional form of the group III–V semiconducting monolayers and may shed light on their potential applications.

## Supplementary Information

Supplementary Information.

## Data Availability

The data used and analysed during the current study is available from the corresponding author on reasonable request.
