# Supplementary material for: First-principles study on the electronic and optical properties of AlSb monolayer
Source: Sci Rep. 2023 Jun 19;13:9925. doi: 10.1038/s41598-023-37081-5 (PMC10279747; doi:10.1038/s41598-023-37081-5)
Supplement: Supplementary file 1 — Supplementary Information. [file 41598_2023_37081_MOESM1_ESM.docx]

Supporting information for

First-principles study on the electronic and optical properties of AlSb monolayer

Mohammad Ali Mohebpour^1^, and Meysam Bagheri Tagani^1^

*^1^ Computational Nanophysics Laboratory (CNL), Department of physics, University of Guilan,
P. O. Box 41335-1914, Rasht, Iran.*

Table S1. Structural parameters of AlSb monolayer including the lattice constant (a), bond lengths (d_1_, d_2_), buckling height (h), and cohesive energy (E_c_).

|  | a (Å) | d_1_ (Å) | d_2_ (Å) | h (Å) | E_c_ (eV/atom) |
| --- | --- | --- | --- | --- | --- |
| This work | 4.29 | 2.71 | 2.86 | 3.96 | 4.49 |
| Ref. [1] | 4.29 | - | - | 3.96 | - |
| Ref. [2] | 4.24 | 2.68 | 2.86 | 3.96 | 5.04 |

Figure S1. Phonon band dispersion of AlSb monolayer, which is free of imaginary frequencies, showing the dynamical stability of the monolayer.

Figure S2. (a) Electronic band structure (PBE, HSE03, and HSE06) along with the total and partial density of states (HSE06) of AlSb monolayer. (b) Orbital-decomposed band structure (HSE06) of AlSb monolayer. (c) Square of wave function (HSE06) at the VBM (bottom panel) and CBM (top panel) of AlSb monolayer in real space.


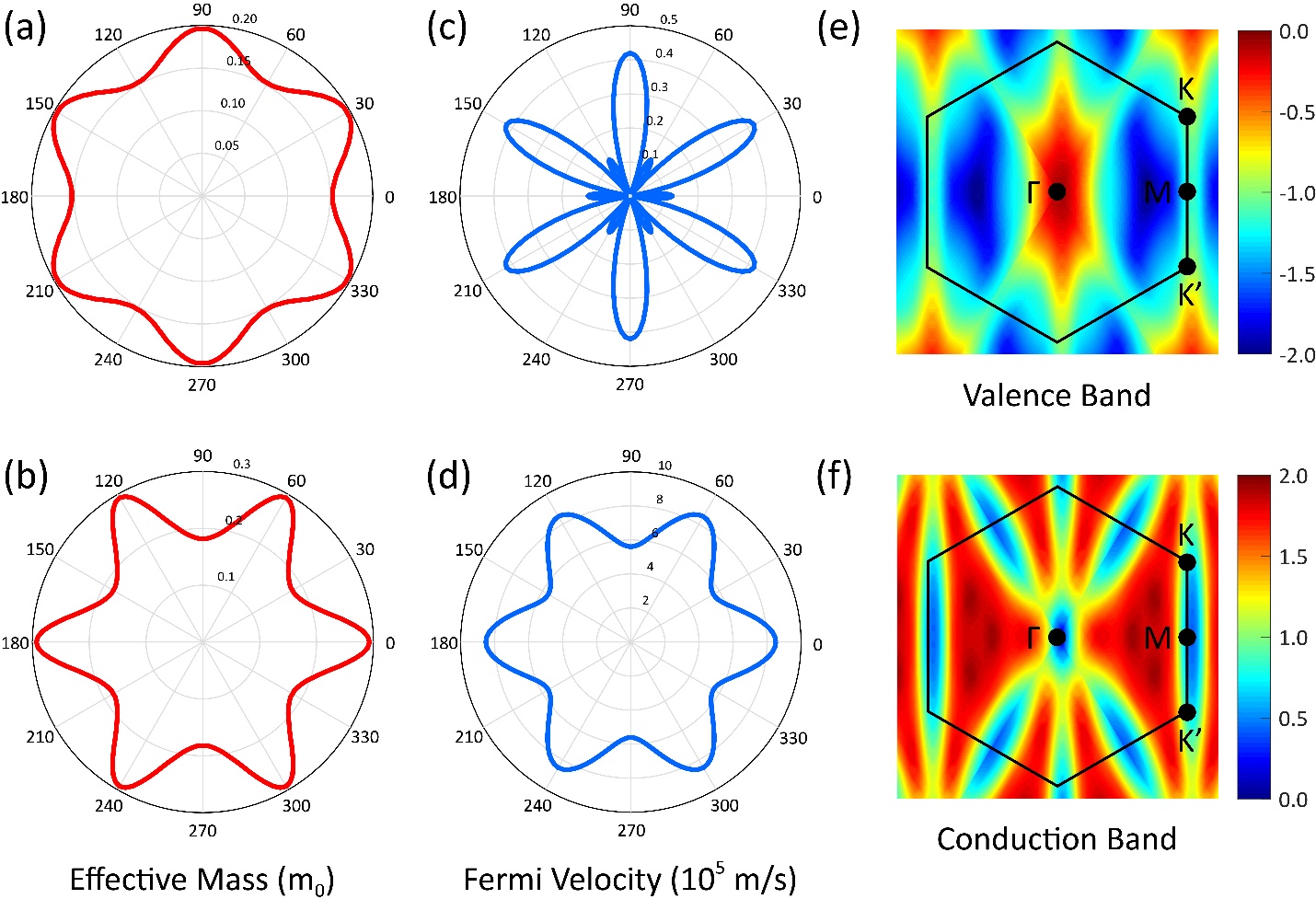


Figure S3. Orientation-dependent effective mass (left panel) and Fermi velocity (middle panel) for holes (a, c) and electrons (b, d) of AlSb monolayer along with the 2D energy map of the highest valence band (e) and lowest conduction band (f). The first BZ is shown by black lines.

Figure S4. (a) Side and (b) perspective views of AlSb monolayer in which the inversion symmetry has been broken by replacing the Al and Sb atoms in one sublayer. (c) Band structure of this conceptual model at the HSE06-SOC level. The separated bands are shown by different colors.

Figure S5. Convergence test for the QP band gap at G_0_W_0_ level of theory as a function of the (a) number of empty bands, (b) vacuum space, and (c) k-point mesh. The convergence tests were performed for two different plane-wave energy cutoffs. (d) Convergence of the QP band gaps achieved from G_i_W_0_ and G_i_W_i_ levels of theory as a function of iteration in the self-consistency.

Figure S6. The electron-hole contribution of the first excitonic peak in reciprocal space. The radius of the circle reflects the magnitude of a particular electron-hole pair contribution.

AlSb monolayer

1.0000000000000000

2.1495386791600000 -3.7231102051399998 0.0000000000000000

2.1495386791600000 3.7231102051399998 0.0000000000000000

0.0000000000000000 0.0000000000000000 20.000000000000000

Al Sb

2 2

Selective dynamics

Direct

0.8333175182611114 0.6666652054973454 0.4560793285742193 T T T

0.1666808427268904 0.3333340851546538 0.5439026631587751 T T T

0.1666238438286101 0.3333538173229513 0.4009264662850427 T T T

0.8333746588613877 0.6666452863440426 0.5990555582349586 T T T

References

[1] L. Qin *et al.*, "Realization of AlSb in the double-layer honeycomb structure: A robust class of two-dimensional material," *ACS nano,* vol. 15, no. 5, pp. 8184-8191, 2021.

[2] A. Bafekry *et al.*, "Novel two-dimensional AlSb and InSb monolayers with a double-layer honeycomb structure: a first-principles study," *Physical Chemistry Chemical Physics,* vol. 23, no. 34, pp. 18752-18759, 2021.
